# Evaluation of dioxin-like polychlorinated biphenyls in fish of the Caspian Sea

**DOI:** 10.1016/j.mex.2020.100803

**Published:** 2020-01-23

**Authors:** Ayub Ebadi Fathabad, Hossein Tajik, Khadijeh Jafari, Edris Hoseinzadeh, Sepideh Sadat Mirahmadi, Gea Oliveri Conti, Mohammad Miri

**Affiliations:** a Department of Food Hygiene and Quality Control, Faculty of Veterinary Medicine, Urmia University, Urmia, Iran; b Environmental Science and Technology Research Center, Department of Environmental Health Engineering, School of Public Health, Shahid Sadoughi University of Medical Sciences, Yazd, Iran; c Environmental Health Engineering Department, Student Research Committee, Saveh University of Medical Sciences, Saveh, Iran; d Department of Food Hygiene and Safety, Faculty of Health, Zanjan University of Medical Sciences and Health Services, Zanjan, Iran; e Environmental and Food Hygiene Laboratory, Department of Medical, Surgical Sciences and Advanced Technologies “G.F. Ingrassia”, University of Catania, Catania, Italy; f Non-Communicable Diseases Research Center, Department of Environmental Health, School of Public Health, Sabzevar University of Medical Sciences & Health Services, Sabzevar, Iran

**Keywords:** Application of evaluation of dioxin-like polychlorinated biphenyls in fish of the Caspian Sea, Dioxins, Polychlorinated biphenyls, Fish

## Abstract

Dioxin-like polychlorinated biphenyls (DL-PCBs) have toxic properties for humans. The innovation of this study was that for the first time in Iran, 12 DL-PCBs concentration in 5 fish species: *Rutilus frisii kutum kanesky*, *Chelon saliens*, *Vimba vimba*, *Cyprinus carpio* and *Oncorhynchus mykiss*, from 5 coastal areas of the Caspian Sea (125 samples), were investigated. DL-PCBs extraction was in accordance to USEPA method 1668 revision A and carry out by chromatography columns modified with silica gel. DL-PCBs concentration were measured by HRGC (Agilent 6890 Series, Agilent Technologies, USA) coupled with HRMS AutoSpec Ultima NT–HRGC/HRMS (Micromass, USA), equipped with the HP-5MS 30 m × 0.25 mm × 0.25 μm column (Agilent Technologies) and helium as carrier gas. The mean concentration of DL-PCBs in samples ranged 232 ± 16–1156 ± 14 pg/g fat, that was in accordance with the Joint FAO/WHO Expert Committee on Food Additives and European Standards. The highest concentration was in *Cyprinus carpio* of Bandar Anzali, and lowest obtained in samples from Chalous. However, based on fish consuming and fish originating from the fishing area the health risk evaluation to estimate the potential consequences of chronic exposure to DL-PCBs for consumers is recommended and effective measure for health risk reduction.

## Graphical abstract

**Figure:**
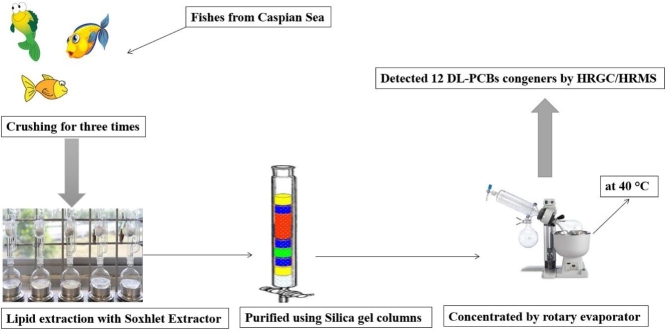

**Specifications Table**

**Table:** 

Subject area:	Environmental Science
More specific subject area:	Food safety
Protocol name:	Application of evaluation of dioxin-like polychlorinated biphenyls in fish of the Caspian Sea
Reagents/tools:	12 DL-PCBs congeners were extracted by chromatography columns modified with silica gel and DL-PCBs were measured using HRGC/HRMS (HRGC (Agilent 6890 Series, Agilent Technologies, USA) coupled with High Resolution Mass Spectrometer AutoSpec Ultima NT–HRGC/HRMS (Micromass, USA), equipped with the HP-5MS 30 m × 0.25 mm × 0.25 μm column (Agilent Technologies) and helium as carrier gas). Meat grinder (Moulinex, Ecully Cedex, France). All of the chemical agent was from Merck, Germany. Internal standard PCB 209 (Sigma-Aldrich, Germany) and Soxhlet Extraction System B-811.
Experimental design:	A total of 125 samples of fish (*Rutilus frisii kutum kanesky*, *Chelon saliens*, *Vimba vimba*, *Cyprinus carpio* and *Oncorhynchus mykiss*) were prepared from 5 coastal areas of the Caspian Sea including Bandar Anzali, Chalous, Rasht, Astara and Bandar Torkaman (25 samples per each city). 12 DL-PCBs congeners were determine in their tissue and then the mentioned parameters above, in abstract section, were analyzed according to the EU and JECFA standards.
Trial registration:	No applicable
Ethics:	No applicable

**Value of the Protocol**

**Table:** 

• Exposure to DL-PCBs can lead to complications due to high resistance, toxic and bioaccumulation in humans and wildlife of DL-PCBs. • Data analysis showed that the mean concentration of DL-PCBs in fish samples were in accordance with the EU and JECFA standards. • Contriling of DL-PCBs in contaminated industries and environmental health to reduse the DL-PCBs concentration in food chain is necessary.

## Description of protocol

### Study area description

The Caspian Sea, in the geographical location of 40 °N and 51 °E, is the largest lake in the world. The average water depth is 187 m and the water volume is 78,200 km^3^. The Caspian Sea is the strategic location for many human needs and activities. Also, Caspian sea is a source of fishing and shrimp fishing for neighboring countries. Annually, 600,000 ton of fish species from this sea are hunted.

### Determination of DL-PCBs concentration in fish samples from Caspian sea

Five common fish species, a total of the 125 fish samples (25 samples from each city), was randomly collected from predetermined stations of 5 location including: Bandar Anzali, Rasht, Chalous, Bandar Torkaman and Astara that placed in cold boxes with ice. In the laboratory, fishes biometrics, were recorded and the muscle tissue was separated about 50 g. Then, the samples were wrapped in aluminum foil and stored at −20 °C until analysis in a dark environment [[1], [2], [3]]. The DL-PCBs extraction was in accordance to USEPA method 1668 revision A [4]. For extraction the DL-PCBs, first, the samples were crushing for three times (Moulinex, Ecully Cedex, France). In each sample, about 50 g of homogenized muscle tissue was combined with 100 g Na_2_SO_4_ and then homogenized at 50 °C for 6 h. In addition, they added about 50 ng of internal standard PCB 209 and using Soxhlet Extractor, the lipid extraction process was carried out. To DL-PCBs extract, hexane and acetone solvents were used in the ratio of 90:10 and about 260 times, repeated extraction [5,6]. The concentration of lipid was determined gravimetrically. 1 g of extracted lipid was dissolved in 10 mL n-hexane, and this diluted extract was used for further analyses. All extracts were purified using silica gel multi-layer absorbent columns [[7], [8], [9]]. The silicates were initially activated [6,10]. The DL-PCBs were passed through the column filled with silica and collected. Finally, the DL-PCBs were eluted through the column by 50 mL *n*-hexane (HPLC grade) and concentrated using a rotary evaporator at 40 °C to reach a final volume of 1 ml for its injection into HRGC/HRMS [6,11]. Also, calibration curve had a good linearity for 1–10 standards from 1−1000 μg/l (R^2^ > 0.99). Mean recoveries were 98 % and 110 % for all 12 congeners of DL-PCBs. The range of 0.03–0.09 pg/g fat were found for limits of quantification (LOQ) in the all DL-PCBs. Thus, the amount of 12 congeners of DL-PCBs (biphenyls NO.0, PCBs NO.77, NO.81, NO.105, NO.114, NO.118, NO.123, No.126, NO.156, NO.157, NO.169, NO.167, NO.189) was determined according to pgTEQ/g fat [12,13]. The toxicity level of the DL-PCBs based on the most toxic known of dioxin compounds, namely, 3,2,7,8 tetra chloro-di benzo-dioxin (TCDD), was considered as the toxic scale of 1 for it and the toxicity of other dioxins-like compounds (DL-PCBs) were compared with it [[14], [15], [16]].

### Statistical design of experiments

**Fig. 1:**
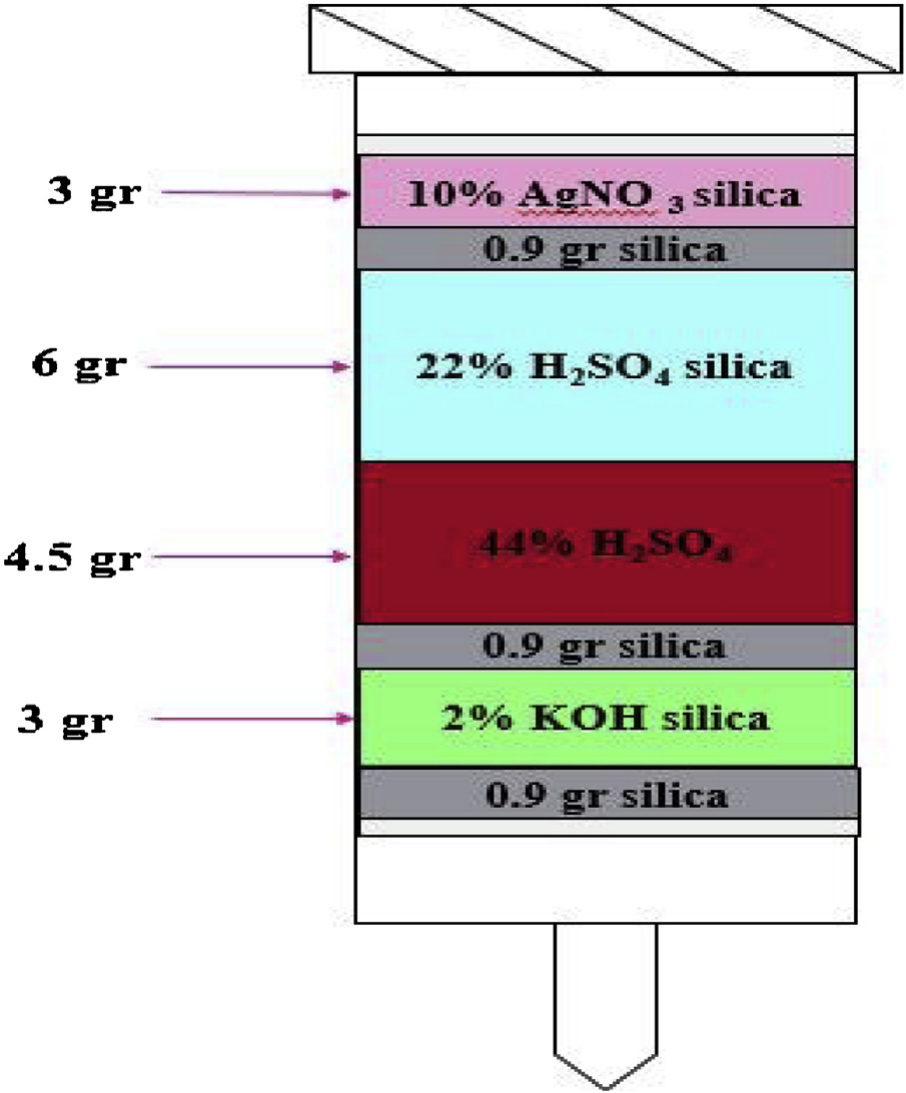
Combination of different layers of silica gel columns used for extraction of DL-PCBs.

**Fig. 2:**
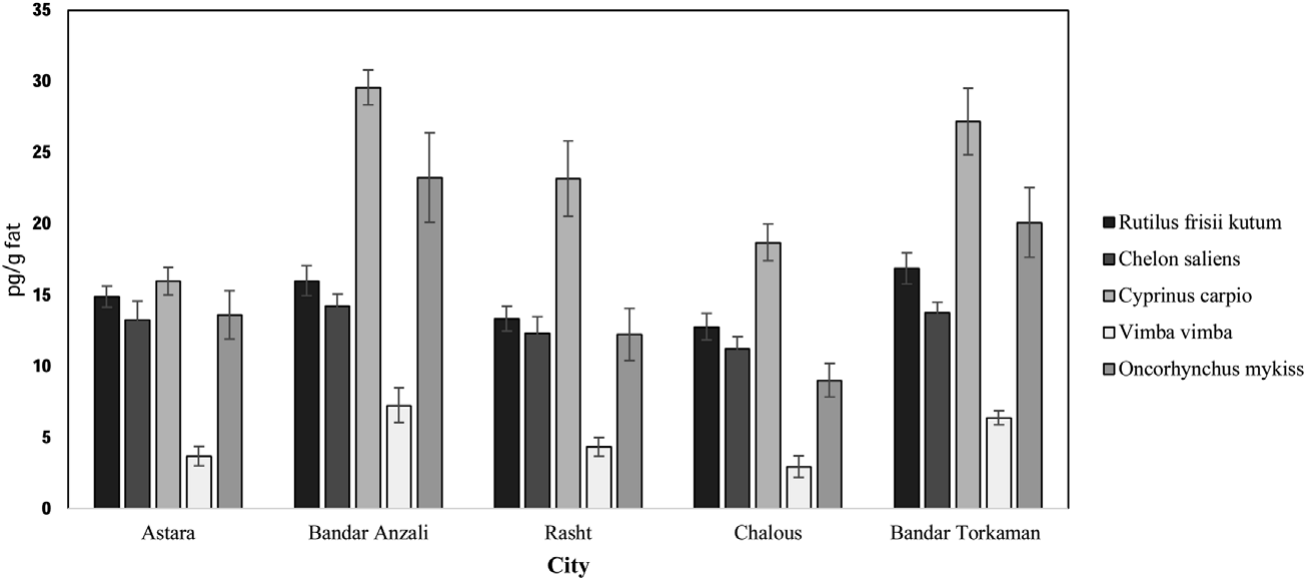
Comparison of PCB77 concentrations in different fish of each city.

**Fig. 3:**
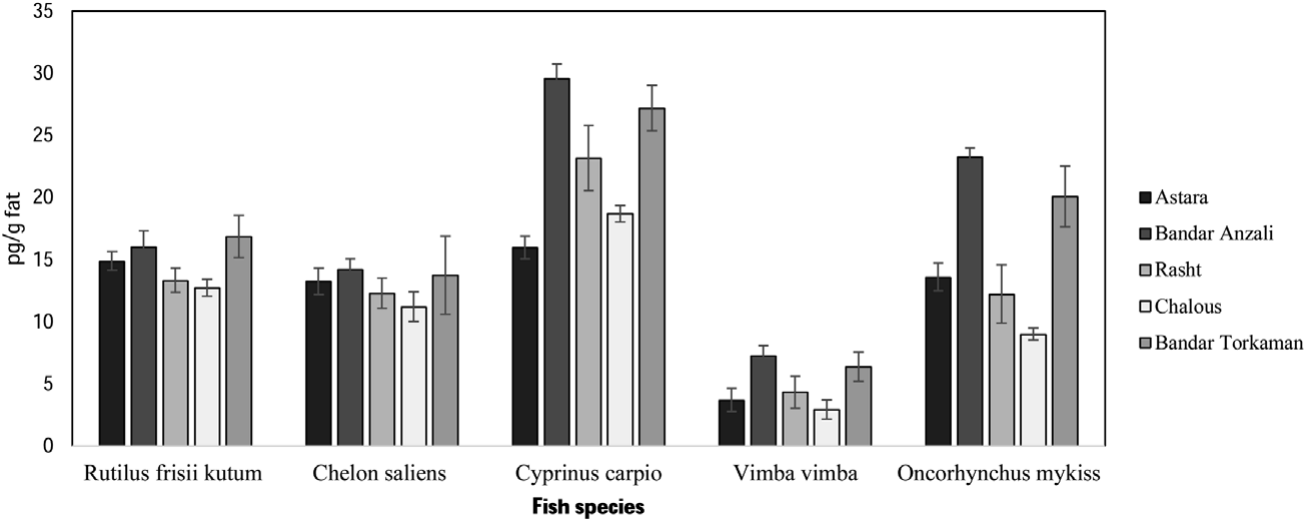
Comparison of PCB77 concentration at different fishing locations.

Data analysis was carried out with SPSS_22_ software (Duncan’s multi-scope test and descriptive statistics). P-value ≤ 0.05 was considered significant. Microsoft Excel version 2016 for plotting calibration curves and basic mathematical calculations. The data presented here deals with DL-PCBs concentration in fish species according fish species and city location. Fig. 1, shown different layers of silica gel columns for extraction of DL-PCBs. Fig. 2, Fig. 3, Table 3, Table 4, Table 5, Table 6, Table 7, Table 8, Table 9, Table 10, Table 11, Table 12, Table 13, Table 14 showed the concentration of DL-PCBs in fish species according type of fish and the city. Table 1, Table 2, Table 3 shown chemical specification of DL-PCBs, Toxicity of various derivatives of DL-PCBs and biometric specifications of different fish samples.

**Table 1:** Chemical specification of DL-PCBs that tested.

Chemical name (IUPAC NO.)	Position of the chlorine atoms	The number of chlorine atoms	Derivatives of DL-PCBs
3,3′,4,4′-Tetrachlorobiphenyl	3,3′,4,4	4	PCB 77
3,4,4′,5-Tetrachlorobiphenyl	3,4,4′,5	4	PCB 81
2,3,3′,4,4′-Pentachlorobiphenyl	2,3,3′,4,4	5	PCB 105
2,3,4,4′,5-Pentachlorobiphenyl	2,3,4,4′,5	5	PCB 114
2,3′,4,4′,5-Pentachlorobiphenyl	2,3′,4,4′,5	5	PCB 118
2,3′,4,4′,5′-Pentachlorobiphenyl	2,3′,4,4′,5	5	PCB 123
3,3′,4,4′,5-Pentachlorobiphenyl	3,3′,4,4′,5	5	PCB 126
2,3,3′,4,4′,5-Hexachlorobiphenyl	2,3,3′,4,4′,5	6	PCB 156
2,3,3′,4,4′,5′-Hexachlorobiphenyl	2,3,3′,4,4′,5	6	PCB 157
2,3′,4,4′,5,5′-Hexachlorobiphenyl	2,3′,4,4′,5,5	6	PCB 167
3,3′,4,4′,5,5′-Hexachlorobiphenyl	3,3′,4,4′,5,5	6	PCB 169
2,3,3′,4,4′,5,5′-Heptachlorobiphenyl	2,3,3′,4,4′,5,5′	7	PCB 189

**Table 2:** Toxicity is equivalent to the various derivatives of DL-PCBs measured.

IUPAC NO.	Type	Structure	WHO-TEF
PCB 77	Non-ortho	3,3′,4,4 TeCB	0.0001
PCB 81	Non-ortho	3,4,4′,5 TeCB	0.0003
PCB 105	Mono-ortho	2,3,3′,4,4 PeCB	0.00003
PCB 114	Mono-ortho	2,3,4,4′,5 PeCB	0.00003
PCB 118	Mono-ortho	2,3′,4,4′,5 PeCB	0.00003
PCB 123	Mono-ortho	2,3′,4,4′,5 PeCB	0.00003
PCB 126	Non-ortho	3,3′,4,4′,5 PeCB	0.1
PCB 156	Mono-ortho	2,3,3′,4,4′,5 HxCB	0.00003
PCB 157	Mono-ortho	2,3,3′,4,4′,5 HxCB	0.00003
PCB 167	Mono-ortho	2,3′,4,4′,5,5 HxCB	0.00003
PCB 169	Non-ortho	3,3′,4,4′,5,5 HxCB	0.03
PCB 189	Mono-ortho	2,3,3′,4,4′,5,5′ HpCB	0.00003

**Table 3:** Biometric Specifications of Different Fish Samples.

City	Bandar-e Torkaman		Chalous		Rasht		Bandar-e Anzali		Astara	
Fish species	Weight (gr)	Length (cm)	Weight (gr)	Length (cm)	Weight (gr)	Length (cm)	Weight (gr)	Length (cm)	Weight (gr)	Length (cm)
*Rutilus frisii kutum*	631.6 ± 77	39.6 ± 1.5	1535.6±88.6	51.2 ± 2.1	1332.8 ± 8.2	50.6 ± 5.3	1297.6 ± 128	49.4 ± 3.3	1386.8 ± 74	48.6 ± 5.2
*Chelon saliens*	790.8 ± 75	47.4 ± 3.2	1148.6±94	49.4 ± 3.1	557 ± 62	43.2 ± 2.3	892.4 ± 2.9	49.6 ± 2.7	149.5 ± 23	25.8 ± 3.4
*Cyprinus carpio*	763.6 ± 48	40.4 ± 4.3	641 ± 31.5	35.8 ± 1.4	1902.4 ± 58	49.2 ± 2.3	3130 ± 185	56 ± 3.5	582.6 ± 63	35.4 ± 3
*Vimba vimba*	50.2 ± 2.4	16.8 ± 0.8	49.5 ± 2.5	16.6 ± 0.6	56.6 ± 3.7	17 ± 1	61.4 ± 1.7	18.5 ± 1.2	51.6 ± 2.4	17.5 ± 1.1
*Oncorhynchus mykis*	1350.8 ± 145	41.2 ± 2.5	1459 ± 87	47.5 ± 4.3	1266.8 ± 95	44.7 ± 3.1	897.8 ± 91	39.5 ± 2.3	878.2 ± 98.6	41.3 ± 4.2

**Table 4:** Comparison of mean concentration of PCB81 (pg/g fat).

Fish species/City	Bandar Torkaman	Chalous	Rasht	Bandar Anzali	Astara
*Rutilus frisii kutum*	1.66 ± 0.47 ^Ab^	1.22 ± 0.27 ^BCb^	0.82 ± 0.10 ^Cb^	1.27 ± 0.36 ^Bb^	0.82 ± 0.06 ^Cb^
*Chelon saliens*	1.24 ± 0.19 ^Ab^	0.71 ± 0.08 ^BCc^	0.52 ± 0.10 ^Dc^	0.78 ± 0.10 ^Bc^	0.61 ± 0.05 ^CDc^
*Cyprinus carpio*	2.48 ± 0.56 ^Aa^	1.81 ± 0.20 ^Ba^	1.25 ± 0.37 ^Ca^	2.12 ± 0.53 ^Aa^	1.09 ± 0.15 ^Ca^
*Vimba vimba*	0.67 ± 0.33 ^Ac^	0.06 ± 0.02 ^Bd^	0.10 ± 0.03 ^Bd^	0.17 ± 0.04 ^Bd^	0.06 ± 0.13 ^Be^
*Oncorhynchus mykiss*	1.17 ± 0.21 ^Ab^	0.88 ± 0.11 ^Bc^	0.20 ± 0.06 ^Cd^	0.33 ± 0.05 ^Cd^	0.21 ± 0.05 ^Cd^

The different small letters indicate a significant difference in the columns and different large letters indicating a significant difference in the row (p ≤ 0.05).

**Table 5:** Comparison of mean concentration of PCB105.

Fish species/City	Bandar Torkaman	Chalous	Rasht	Bandar Anzali	Astara
*Rutilus frisii kutum*	225.76 ± 8.82 ^Cb^	182.23 ± 8.42 ^Db^	237.40 ± 7.10 ^Bb^	257.21 ± 6.61 ^Ab^	228.29 ± 8.82 ^BCb^
*Chelon saliens*	180.12 ± 10.86 ^Cc^	152.09 ± 6.12 ^Dc^	210.78 ± 8.70 ^Bc^	223.54 ± 12.25 ^Ac^	212.21 ± 10.86 ^ABc^
*Cyprinus carpio*	267.89 ± 15.29 ^Ba^	201.62 ± 10.26 ^Da^	250.54 ± 8.75 ^Ca^	309.84 ± 15.27 ^Aa^	242.67 ± 8.64 ^Ca^
*Vimba vimba*	119.84 ± 7.86 ^Be^	78.76 ± 8.67 ^De^	108.80 ± 6.01 ^Ce^	129.89 ± 8.30 ^Ae^	103.92 ± 4.72 ^Ce^
*Oncorhynchus mykiss*	156.38 ± 15.01 ^Ad^	100.24 ± 9.45 ^Cd^	136.23 ± 9.22 ^Bd^	163.53 ± 7.44 ^Ad^	123.08 ± 55.43 ^Bd^

The different small letters indicate a significant difference in the columns and different large letters indicating a significant difference in the row (p ≤ 0.05).

**Table 6:** Comparison of mean concentration of PCB114.

Fish species/City	Bandar Torkaman	Chalous	Rasht	Bandar Anzali	Astara
*Rutilus frisii kutum*	20.61 ± 0.83 ^ABb^	13.66 ± 0.58 ^Db^	19.29 ± 1.55 ^BCb^	21.16 ± 1.16 ^Ab^	18.08 ± 1.09 ^Ca^
*Chelon saliens*	17.89 ± 0.73 ^Ac^	10.88 ± 0.48 ^Cc^	15.25 ± 1.02 ^Bc^	17.76 ± 0.69 ^Ac^	15.59 ± 1.10 ^Bb^
*Cyprinus carpio*	24.30 ± 0.79 ^Ba^	15.78 ± 1.09 ^Ea^	21.87 ± 1.18 ^Ca^	27.46 ± 1.22 ^Aa^	18.94 ± 0.69 ^Da^
*Vimba vimba*	10.19 ± 1.00 ^Ae^	4.22 ± 0.66 ^De^	8.42 ± 1.05 ^Bd^	9.86 ± 0.70 ^Ae^	6.79 ± 1.34 ^Cd^
*Oncorhynchus mykiss*	14.34 ± 0.77 ^AB^	7.83 ± 0.81 ^Dd^	13.85 ± 0.90 ^Bc^	15.43 ± 0.76 ^Ad^	10.38 ± 1.18 ^Cc^

The different small letters indicate a significant difference in the columns and different large letters indicating a significant difference in the row (p ≤ 0.05).

**Table 7:** Comparison of mean concentration of PCB118 (pg/g fat).

Fish species/City	Bandar Torkaman	Chalous	Rasht	Bandar Anzali	Astara
*Rutilus frisii kutum*	318.37 ± 6.01 ^Bb^	238.99 ± 6.21 ^Db^	289.39 ± 6.88 ^Cb^	350.24 ± 5.26 ^Ab^	288.92 ± 6.05 ^Cb^
*Chelon saliens*	308.11 ± 25.35 ^Ab^	219.57 ± 4.15 ^Cc^	257.64 ± 5.65 ^Bc^	315.60 ± 7.28 ^Ac^	258.41 ± 7.96 ^Bc^
*Cyprinus carpio*	339.44 ± 6.76 ^Ba^	261.87 ± 8.11 ^Ea^	320.14 ± 9.80 ^Ca^	367.50 ± 8.30 ^Aa^	302.81 ± 8.31 ^Da^
*Vimba vimba*	167.37 ± 5.32 ^Bd^	91.48 ± 5.63 ^Ee^	152.22 ± 8.28 ^Ce^	189.12 ± 7.22 ^Ae^	136.49 ± 6.20 ^De^
*Oncorhynchus mykiss*	235.75 ± 5.89 ^Bc^	181.23 ± 4.27 ^Dd^	209.64 ± 3.99 ^Cd^	252.71 ± 10.95 ^Ad^	241.83 ± 3.67 ^Bd^

The different small letters indicate a significant difference in the columns and different large letters indicating a significant difference in the row (p ≤ 0.05).

**Table 8:** Comparison of mean concentration of PCB123.

Fish species/City	Bandar Torkaman	Chalous	Rasht	Bandar Anzali	Astara
*Rutilus frisii kutum*	140.72 ± 11.20 ^Aab^	98.69 ± 4.10 ^Cb^	101.93 ± 2.81 ^Cb^	123.12 ± 3.76 ^Bb^	80.52 ± 1.45 ^Db^
*Chelon saliens*	131.06 ± 4.78 ^Ab^	71.57 ± 5.40 ^Ec^	91.80 ± 3.16 ^Cc^	112.54 ± 5.14 ^Bc^	80.83 ± 4.28 ^Db^
*Cyprinus carpio*	149.34 ± 5.70 ^Ba^	122.83 ± 4.37 ^Ca^	118.09 ± 4.29 ^Ca^	170.56 ± 6.^30Aa^	97.32 ± 5.09 ^Da^
*Vimba vimba*	74.21 ± 7.05 ^ABd^	39.76 ± 5.02 ^Ce^	69.34 ± 5.93 ^Bd^	80.18 ± 5.20 ^Ae^	43.66 ± 4.46 ^Cd^
*Oncorhynchus mykiss*	108.41 ± 8.08 ^Ac^	50.29 ± 5.17 ^Cd^	73.50 ± 7.65 ^Bd^	99.50 ± 3.42 ^Ad^	56.87 ± 10.41 ^Cc^

The different small letters indicate a significant difference in the columns and different large letters indicating a significant difference in the row (p ≤ 0.05).

**Table 9:** Comparison of mean concentration of PCB126.

Fish species/City	Bandar Torkaman	Chalous	Rasht	Bandar Anzali	Astara
*Rutilus frisii kutum*	7.24 ± 0.57 ^ABb^	6.41 ± 0.54 ^BCb^	6.38 ± 0.82 ^BCb^	8.09 ± 0.73 ^Ab^	6.13 ± 0.67 ^Cb^
*Chelon saliens*	5.00 ± 0.71 ^Bc^	4.86 ± 0.49 ^Bc^	5.10 ± 0.54 ^Bc^	6.21 ± 0.42 ^Ac^	5.85 ± 0.57 ^Ab^
*Cyprinus carpio*	8.98 ± 1.06 ^Ba^	7.30 ± 0.47 ^Ca^	8.18 ± 0.48 ^BCa^	10.23 ± 0.69 ^Aa^	7.34 ± 0.50 ^Ca^
*Vimba vimba*	1.71 ± 0.73 ^Bd^	1.13 ± 0.30 ^BCe^	1.34 ± 0.45 ^Be^	2.34 ± 0.29 ^Ae^	0.59 ± 0.15 ^Cd^
*Oncorhynchus mykiss*	4.22 ± 0.42 ^Ac^	2.89 ± 0.33 ^Bd^	4.00 ± 0.38 ^Ad^	3.92 ± 0.55 ^Ad^	1.95 ± 0.64 ^Cc^

The different small letters indicate a significant difference in the columns and different large letters indicating a significant difference in the row (p ≤ 0.05).

**Table 10:** Comparison of mean concentration of PCB156.

Fish species/City	Astara	Bandar Anzali	Rasht	Chalous	Bandar Torkaman
*Rutilus frisii kutum*	32.91 ± 0.90 ^Db^	52.76 ± 2.37 ^Ab^	44.38 ± 2.78 ^Bb^	41.35 ± 2.69 ^Cb^	51.72 ± 1.44 ^Aab^
*Chelon saliens*	26.46 ± 1.54 ^Cc^	49.68 ± 1.10 ^Ac^	35.47 ± 2.45 ^Bc^	36.83 ± 1.30 ^Bc^	49.06 ± 2.47 ^Ab^
*Cyprinus carpio*	43.28 ± 1.49 ^Ca^	57.80 ± 4.17 ^Aa^	52.18 ± 4.02 ^Ba^	47.45 ± 1.77 ^Ca^	54.29 ± 4.16 ^ABa^
*Vimba vimba*	3.80 ± 0.51 ^CDe^	11.81 ± 0.65 ^Ae^	4.16 ± 1.18 ^Ce^	3.08 ± 0.74 ^De^	9.06 ± 0.50 ^Bd^
*Oncorhynchus mykiss*	22.10 ± 1.30 ^Dd^	38.60 ± 1.45 ^Bd^	40.07 ± 1.38 ^ABd^	25.66 ± 2.04 ^Cd^	41.96 ± 1.91 ^Ac^

The different small letters indicate a significant difference in the columns and different large letters indicating a significant difference in the row (p ≤ 0.05).

**Table 11:** Comparison of mean concentration of PCB157.

Fish species/City	Bandar Torkaman	Chalous	Rasht	Bandar Anzali	Astara
*Rutilus frisii kutum*	38.21 ± 1.19 ^Ba^	20.80 ± 1.20 ^Eb^	31.73 ± 0.81 ^Cb^	41.91 ± 0.93 ^Ab^	23.69 ± 1.01 ^Db^
*Chelon saliens*	39.57 ± 1.47 ^Aa^	18.27 ± 0.75 ^Cc^	24.84 ± 1.19 ^Bc^	38.18 ± 1.43 ^Ac^	17.48 ± 0.90 ^Cc^
*Cyprinus carpio*	37.96 ± 1.32 ^Ca^	31.74 ± 1.21 ^Da^	41.81 ± 0.55 ^Ba^	46.42 ± 1.04 ^Aa^	31.31 ± 1.28 ^Da^
*Vimba vimba*	1.73 ± 0.42 ^Cc^	0.82 ± 0.16 ^Ed^	1.14 ± 0.31 ^Dd^	8.05 ± 0.63 ^Ae^	2.28 ± 0.43 ^Bd^
*Oncorhynchus mykiss*	24.16 ± 1.67 ^Cb^	18.34 ± 0.84 ^Dc^	27.90 ± 1.11 ^Bc^	30.42 ± 1.30 ^Ad^	18.16 ± 0.90 ^Dc^

**Table 12:** Comparison of mean concentration of PCB167.

Fish species/City	Bandar Torkaman	Chalous	Rasht	Bandar Anzali	Astara
*Rutilus frisii kutum*	87.67 ± 2.85 ^Bb^	60.55 ± 1.68 ^Eb^	82.38 ± 0.78 ^Cb^	98.41 ± 2.70 ^Ab^	75.11 ± 1.25 ^Db^
*Chelon saliens*	78.34 ± 1.59 ^Bc^	50.52 ± 1.61 ^Ec^	75.30 ± 1.01 ^Cc^	91.46 ± 2.09 ^Ac^	68.54 ± 1.70 ^Dc^
*Cyprinus carpio*	103.45 ± 1.81 ^Ba^	74.57 ± 2.23 ^Ea^	89.07 ± 2.50 ^Ca^	113.76 ± 2.83 ^Aa^	80.98 ± 1.90 ^Da^
*Vimba vimba*	14.60 ± 1.04 ^Be^	9.14 ± 0.64 ^De^	15.24 ± 0.97 ^Be^	20.76 ± 1.35 ^Ae^	11.37 ± 1.46 ^Ce^
*Oncorhynchus mykiss*	51.18 ± 3.09 ^Bd^	32.21 ± 1.20 ^Ed^	48.04 ± 2.54 ^Cd^	70.52 ± 1.77 ^Ad^	36.30 ± 1.02 ^Dd^

The different small letters indicate a significant difference in the columns and different large letters indicating a significant difference in the row (p ≤ 0.05).

**Table 13:** Comparison of mean concentration of PCB169.

Fish species/City	Bandar Torkaman	Chalous	Rasht	Bandar Anzali	Astara
*Rutilus frisii kutum*	0.61 ± 0.02 ^Cb^	0.57 ± 0.06 ^Cb^	0.87 ± 0.08 ^Ba^	1.20 ± 0.35 ^Ab^	0.73 ± 0.10 ^BCb^
*Chelon saliens*	0.57 ± 0.06 ^BCb^	0.43 ± 0.04 ^Cc^	0.70 ± 0.11 ^ABb^	0.78 ± 0.07 ^Ac^	0.71 ± 0.26 ^ABb^
*Cyprinus carpio*	1.19 ± 0.47 ^Ba^	0.82 ± 0.09 ^Ca^	0.92 ± 0.22 ^BCa^	1.65 ± 0.17 ^Aa^	0.99 ± 0.12 ^BCa^
*Vimba vimba*	0.04 ± 0.02 ^ABc^	0.02 ± 0.03 ^Bd^	0.04 ± 0.03 ^ABc^	0.06 ± 0.03 ^Ad^	0.05 ± 0.03 ^ABc^
*Oncorhynchus mykiss*	0.72 ± 0.12 ^Ab^	0.43 ± 0.02 ^Bc^	0.69 ± 0.07 ^Ab^	0.68 ± 0.20 ^Ac^	0.67 ± 0.09 ^Ab^

**Table 14:** Comparison of mean concentration of PCB189.

Fish species/City	Bandar Torkaman	Chalous	Rasht	Bandar Anzali	Astara
*Rutilus frisii kutum*	13.94 ± 1.13 ^ABb^	11.69 ± 0.71 ^Cb^	14.40 ± 1.70 ^ABb^	15.03 ± 0.89 ^Ab^	13.14 ± 0.89 ^BCb^
*Chelon saliens*	12.04 ± 0.88 ^Bc^	9.16 ± 0.75 ^Dc^	13.40 ± 1.00 ^Ab^	13.28 ± 0.85 ^Ac^	10.43 ± 0.86 ^Cc^
*Cyprinus carpio*	17.12 ± 0.92 ^Ba^	15.02 ± 0.67 ^Ca^	18.09 ± 0.47 ^ABa^	19.36 ± 1.44 ^Aa^	15.28 ± 1.20 ^Ca^
*Vimba vimba*	1.96 ± 0.26 ^Bd^	1.00 ± 0.14 ^Cd^	1.12 ± 0.25 ^Cc^	3.15 ± 0.28 ^Ad^	0.98 ± 0.14 ^Ce^
*Oncorhynchus mykiss*	13.46 ± 1.03 ^Bb^	9.34 ± 0.62 ^Cc^	14.02 ± 0.35 ^Bb^	15.50 ± 0.77 ^Ab^	9.04 ± 0.55 ^Cd^

The different small letters indicate a significant difference in the columns and different large letters indicating a significant difference in the row (p ≤ 0.05).
